# Asymmetries in ionization of atomic superposition states by ultrashort laser pulses

**DOI:** 10.1038/s41598-020-73196-9

**Published:** 2020-09-30

**Authors:** J. Venzke, A. Becker, A. Jaron-Becker

**Affiliations:** JILA and Department of Physics, University of Colorado, Boulder, CO 80309-0440 USA

**Keywords:** Attosecond science, Atomic and molecular interactions with photons

## Abstract

Progress in ultrafast science allows for probing quantum superposition states with ultrashort laser pulses in the new regime where several linear and nonlinear ionization pathways compete. Interferences of pathways can be observed in the photoelectron angular distribution and in the past they have been analyzed for atoms and molecules in a single quantum state via anisotropy and asymmetry parameters. Those conventional parameters, however, do not provide comprehensive tools for probing superposition states in the emerging research area of bright and ultrashort light sources, such as free-electron lasers and high-order harmonic generation. We propose a new set of generalized asymmetry parameters which are sensitive to interference effects in the photoionization and the interplay of competing pathways as the laser pulse duration is shortened and the laser intensity is increased. The relevance of the parameters is demonstrated using results of state-of-the-art numerical solutions of the time-dependent Schrödinger equation for ionization of helium atom and neon atom.

## Introduction

Ultrabright light sources such as free-electron lasers^1^ and table-top laser systems based on high-order harmonic generation^2,3^ deliver high-intensity pulses of few- or sub-femtosecond duration. Nowadays, laser pulses with a duration of a few tens of attoseconds have been achieved experimentally^4,5^. Isolated attosecond pulses or trains of attosecond pulses have been generated from the vacuum ultraviolet to the soft X-ray wavelength regime and the polarization of such pulses can nowadays be controlled. This recent progress in ultrafast laser pulse technology makes it possible to probe, steer and control the dynamics of electrons in atoms, molecules and solids^6–11^. To name a few examples, the time-resolved measurement of the electron emission in the photoelectric effect has been realized^12–16^. Using an isolated attosecond pulse or a train of extreme-ultraviolet attosecond pulses to ionize an atom along with an infrared laser field interferograms have been measured to obtain information about phases of electron wavepackets, an important milestone towards reconstructing the wavefunctions of atoms^17,18^. By extracting the phase and amplitude via application of photoelectron spectroscopy recently the birth of a photoelectron through a Fano resonance has been observed on the attosecond time scale^19^.

Probing atoms and molecules in their ground or excited states with ultrashort laser pulses opens a new regime where several linear and nonlinear ionization pathways compete and interfere^20–27^. For example, it has been shown how the competition between resonant and nonresonant pathways depends on the pulse width^20^. An important observable are photoelectron angular distributions (PAD), which are measured by detecting the probability for emission of the electron from the target in different directions. Since PADs are determined by the amplitudes and phases of the partial waves of all pathways contributing to the emission, they are practical means to identify the different contributing pathways. A characteristic signature of such interferences are asymmetries in the emission of the photoelectron^28^. In the simple case of photoionization from a single state, anisotropy and asymmetry parameters have been used in the past to identify and analyze interesting physical effects. A significant circular dichroism via the asymmetry in the forward-backward electron emission from bromocamphor molecules induced by circularly polarized light has been identified^29^. Observation of the breakdown of the symmetry in the photoelectron emission of argon has been shown in the region of the Cooper minimum^30^. Interferences between resonant and non-resonant pathways^20^ or direct and autoionizing channels^31^ can be identified via anisotropy and asymmetry parameters. Other examples can be found in double photoionization^32^ or molecular vibrations and chirality^33^ and applications range from studies of coherent control^34^ to the characterization of ultrashort laser pulses^35^.

While studies of quantum systems in a single state are important, very interesting physics arises from the systems in superposition states. Nowadays the most prominent example of a two-level quantum mechanical system is a qubit with its important applications in quantum computation and quantum simulations^36^. Yet, also the internal motion of quantum mechanical systems, whether it is rotational, vibrational or electronic, is determined via superposition states. In ultrafast science the observation and resolution of such dynamics and, hence, the observation of atoms or molecules in superposition states has always played a central role. Currently, it is the superposition of atomic or molecular electronic states and the related attosecond electron dynamics that is the focus of studies in the field^18,37–39^. Perhaps the simplest case of such dynamics is a helium atom in a superposition of 1*s* and $2p_1$ state which results in a wavepacket rotating in a plane around the nucleus with a period of $\sim 200$ attoseconds. The dynamics in such quantum systems in superposition states can be probed via ionization with an ultrashort laser pulse. Unlike for the ionization of a quantum system prepared in a single state, e.g. the ground state, conventional anisotropy and asymmetry parameters fail to provide comprehensive tools for the analysis of photoionization from atomic superposition states. For example, the simplest case of a competition between one- and two-photon ionization processes can be analyzed using asymmetry parameters if the atom is prepared in the ground state^20–27^. In contrast, these analysis tools are either not applicable or do not provide a straightforward interpretation for the same processes if the atom is in the superposition of two states. Thus, an extension of the toolbox for the characterization of the states and the identification of competing pathways is desirable. In this paper, we propose a new set of generalized asymmetry parameters which are sensitive to interference effects in the photoionization of atomic systems in superposition states. As we will show these new parameters can be used to identify the interplay of competing linear and nonlinear pathways at low and high intensities, as well as at ultrashort pulse durations. The application and relevance of the parameters is tested using state-of-the-art numerical solutions of the time-dependent Schrödinger equation. Our method provides a new approach to the analysis of experiments dedicated to resolving attosecond electron dynamics.

## Generalized asymmetry parameters

**Figure 1:**
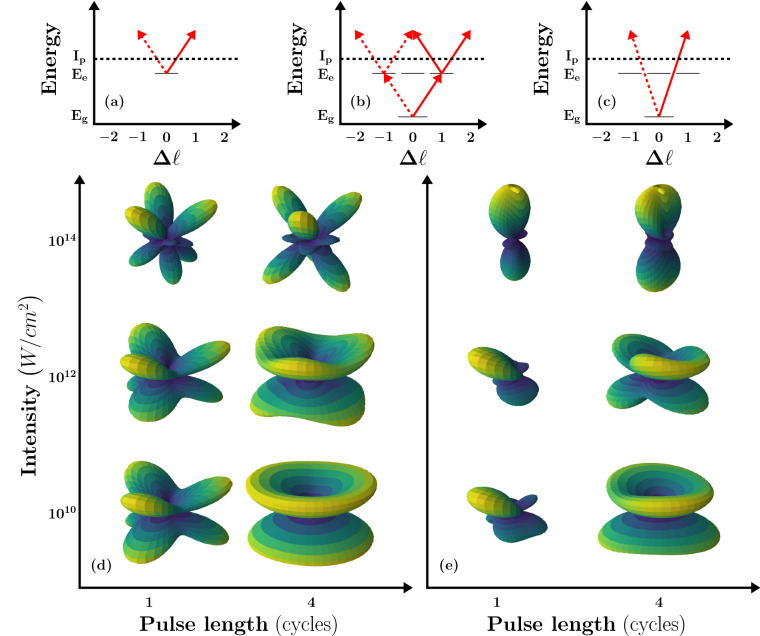
(**a**–**c**) Ionization pathways effective in different intensity and pulse length regimes. (**d**) Photoelectron angular distributions for ionization of neon atom, prepared in $2p_{-1}-3d_2$ superposition, as function of intensity and pulse length. (**e**) The same as (**d**) including additionally ionization from the $2p_0$ and $2p_1$ states.

We consider a prototypical example in ultrafast science and, more general, in atomic physics, which is schematically shown in Fig. 1. An atom in a superposition of two quantum states with different magnetic quantum numbers *m*, say the ground (g) and an excited (e) state is probed via ionization by an ultrashort linearly polarized laser pulse. The photoelectron emission is induced by a pulse with central frequency $\omega$ tuned to the energy gap of the two states. The broad energy spectrum of an ultrashort Gaussian pulse is schematically depicted in the panels of Fig. 1 centered about the energy of the excited state. There are three competing pathways leading to photoelectron emission with energy $2\omega - |E_g|$, where $E_g$ is the ground state energy of the atom: (a) absorption of one photon at $\omega$ from the excited state, (b) absorption of two photons with sum frequency $2\omega$ from the ground state and (c) absorption of one photon at $2\omega$ from the ground state. While the ionization from the excited state (a) is the dominant pathway at low peak intensities and long pulse duration, the transitions from the ground state will interfere at higher peak intensities [two-photon process, (b)] and if the bandwidth of the pulse is broad enough [i.e., the pulse duration is sufficiently short, (c)]. The exemplary results obtained from the solutions of the time-dependent Schrödinger equation in Fig. 1 for the interaction of neon atom, prepared in the superposition of $2p_{-1}$- and $3d_2$-states (d) or prepared in the same superposition but including additionally ionization from the $2p_0$ and $2p_1$ ground states (e), with a linearly polarized laser pulse show that the photoelectron angular distributions (PADs) vary significantly as function of pulse duration and peak intensity. The forward–backward asymmetry, often used in the past, cannot be applied to identify the impact of the different pathways in these PADs. Therefore, a new set of asymmetry parameters is needed for photoionization from superposition states with ultrashort laser pulses.

### Definition

We start by expanding the PAD as a coherent sum of spherical harmonics, assuming the atom is ionized by a linearly polarized pulse aligned along the *z*-axis as

1 $$\begin{aligned} P(\theta ,\phi ) = \left| \sum _\ell C_{\ell }^{m_g} Y_\ell ^{m_g}(\theta ,\phi )+C_{\ell }^{m_e} Y_\ell ^{m_e}(\theta ,\phi )\right| ^2 \end{aligned}$$

where $m_g$ ($m_e$) are the magnetic quantum numbers of the ground (excited) state in the superposition, $C_\ell ^m$ is the complex amplitude and $Y_\ell ^m(\theta ,\phi )$ is the spherical harmonic. The asymmetry in the PADs due to the interference between different channels is related to the relative phase and amplitude of the spherical harmonics. For each spherical harmonic, the sign of the phase is symmetric (asymmetric) across the *xy*-plane when $\ell +m$ is even (odd), while in the *xy*-plane the phase is proportional to $e^{im\phi }$.

**Figure 2:**
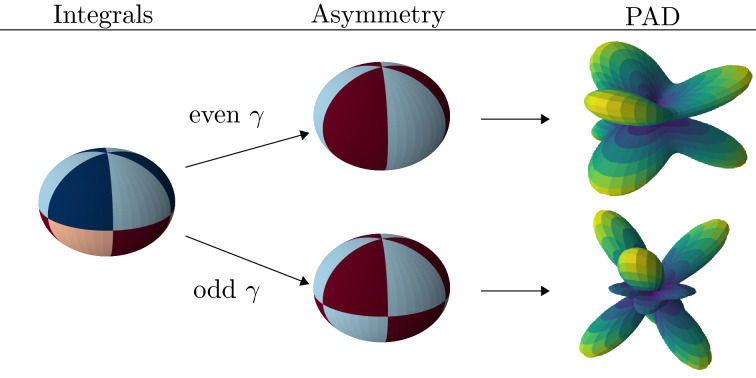
Conceptual illustration of GAPs (for $\Delta m=3$). Left: Integrals $I_\pm ^{even/odd}$, Eqs. (3, 4), are defined over regions of constructive and destructive inferference, indicated by different colors (dark blue, light blue, dark red, light red), in *xy*-plane. Middle: GAPs are constructed based on the parity of the parameter $\gamma = l_e+m_e+l_g+m_g+N_p$ from the integrals over the regions denoted by a certain color (light blue, dark red). Right: Examplary PADs displaying the asymmetry captured by the parameters for even and odd $\gamma$.

In the *xy*-plane there are regions of destructive and constructive interference between the transition amplitudes from the ground and excited state, as illustrated on the left of Fig. 2. The regions can be labeled by

2 $$\begin{aligned} c_i = {\lfloor }{\frac{(\phi -\phi _0)\Delta m}{\pi }}{\rfloor } \end{aligned}$$

where ${\lfloor }{}{\rfloor }$ is the floor function, $\phi _0$ is a reference angle, and $\Delta m=|m_g-m_e|$ ($\Delta m = 3$ in Fig. 2,^40^). Setting $\phi _0$ at an angle where the interference switches from constructive to destructive, regions of destructive interference signal are labeled by even $c_i$ while odd $c_i$ denote regions of constructive interference. Next we define the following integrals:

3 $$\begin{aligned} I^{even}_{\pm }&= \sum _{even \; c_i} \int P(\theta ,\phi )\; d\Omega _{c_i}^{\pm } \end{aligned}$$

4 $$\begin{aligned} I^{odd}_{\pm }&= \sum _{odd \; c_i} \int P(\theta ,\phi )\; d\Omega _{c_i}^{\pm } \end{aligned}$$

where $d\Omega _{c_i}$ is the solid angle for the region $c_i$ in the positive ($z>0$) or negative ($z<0$) hemisphere with respect to the *xy*-plane. In the example in Fig. 2 (left) each integral represents the total photoelectron signal in the regions of a specific color (dark blue, light blue, dark red, light red). We now define general asymmetry parameters (GAPs) that account for the relative difference in the regions of constructive and destructive interference:

5 $$\begin{aligned} A_{p}^{\Delta m} \equiv {\left\{ \begin{array}{ll} \left| \frac{(I^{even}_+ + I^{even}_-) - (I^{odd}_++I^{odd}_-)}{I^{even}_++I^{even}_- + I^{odd}_++I^{odd}_-}\right| _{max(\phi _0)} &{} \text {even} \; \gamma \\ \left| \frac{(I^{even}_++I^{odd}_-) - (I^{odd}_++I^{even}_-)}{I^{even}_++I^{even}_- + I^{odd}_++I^{odd}_-}\right| _{max(\phi _0)} &{} \text {odd} \; \gamma \end{array}\right. } \end{aligned}$$

where $\gamma = \ell _e + m_e + \ell _g + m_g + N_p$ with $\ell _g$ ($\ell _e$) are the quantum numbers of the ground (excited) state in the superposition, and $p=(\gamma \text { mod } 2)$ is the parity of $\gamma$. Each parameter $A_{p}^{\Delta m}$ is related to a certain total number of absorbed photons, $N_p = N_{g}+N_{e}$, where $N_g$ ($N_e$) is the number of photons absorbed in the transition from the ground (excited) state. We note that GAPs cannot only be defined for ionization of superposition states with a linearly polarized laser pulse along the *z*-axis, but the definition can be extended, for example, to ionization with a circularly polarized ionizing laser pulse in the *xy*-plane. In that case $\gamma =\ell _e + m_e + \ell _g + m_g$, the number of photons is not included as both $\ell$ and *m* change by 1 for each photon absorbed. Using $\Delta m = |(m_g \pm N_{pg}) - (m_e \pm N_{pe})|$ for right (+) and left (-) handed circular polarized probe pulses, processes with different number of photons involved can be studied by analyzing the signals with different $\Delta m$. Here, in the further discussion and applications we however focus on the case of a linearly polarized probe pulse.

To exemplify the significance of the asymmetry parameters we consider the ionization of Ne atom, initially prepared in the $2p_{-1}-3d_2$ superposition (i.e., $\ell _g = 1$, $m_g = -1$, $\ell _e = 2$, $m_e = 2$; and, hence, $l_g + m_g + l_e + m_e = 4$), by an ultrashort intense laser pulse. As discussed at the outset, we expect interferences between two kind of pathways depending on the peak intensity and the pulse duration. For an ultrashort pulse at low intensities one-photon transitions from the ground (Fig. 1c, $N_g = 1$) and the excited states (Fig. 1a, $N_e = 1$) will interfere, with $N_p = N_g + N_e = 2$, $\gamma = 6$ and $p=0$. An examplary PAD, obtained via numerical TDSE solution in the relevant intensity and pulse duration regimes, is shown at the top of the right column in Fig. 2. For even $\gamma$ the corresponding parameter $A_0^3$ relates to the difference between the total photoelectron signals in the dark red and light blue shaded regions, as depicted at the top of the middle column of Fig. 2. Comparison with the PAD shows that this difference indeed accounts for the asymmetry in the PAD induced by the interference of the transition amplitudes for the one-photon processes from the ground and the excited states. In contrast, due to the dependence of multiphoton transition amplitudes on the intensity of the pulse at longer pulse duration and higher intensities it is expected that the one-photon transition from the excited state (Fig. 1a, $N_e = 1$) interferes with the two-photon absorption from the ground state (Fig. 1b, $N_g = 2$), giving rise to $N_p = 3$, $\gamma = 7$ and $p=1$ for ionization of Ne($2p_{-1}-3d_2$). The regions relevant in the calculation of the asymmetry parameter $A_1^3$ and the corresponding exemplary PAD for the Ne atom are presented in the bottom row of Fig. 2. The comparison indicates the significance of the asymmetry parameter $A_1^3$ for the detection of the interference at high intensities.

### Application via numerical simulations

For the application of the GAPs we have considered certain superpositions of two atomic states which are first prepared by a pump pulse and then probed by a linearly polarized pulse at a set time delay such that the relative phase between the two states is determined. In the calculations we have therefore simulated the interaction with the probe pulse only by using numerical solutions of the time-dependent Schrödinger equation (TDSE) for the interaction of a electron in a single-active-electron (SAE) potential with the electric field ${\mathbf {E}}$ of a linearly polarized laser pulse (aligned along the quantization ${{\hat{z}}}$-axis) in dipole approximation and length gauge (we use Hartree atomic units $e = m_e = \hslash =1$):

6 $$\begin{aligned} i\frac{\partial }{\partial t}\Psi ({\mathbf {r}},t) = \left[ -\frac{\nabla ^2}{2} - {\mathbf {E}}(t) \cdot {\mathbf {z}} + V(r)\right] \Psi ({\mathbf {r}},t). \end{aligned}$$

To ensure that the electric field integrates to zero^35^, we set the vector potential as:

7 $$\begin{aligned} A_z(t)= & {} A_0 \exp \left( -\ln (2)\left( \frac{2(t-\tau _0)}{T}\right) ^2\right) \nonumber \\&\times \sin \left( \omega _A(t-\tau _0) + \varphi \right) \end{aligned}$$

with $A_0 = \frac{c\sqrt{I_0}}{\omega _A}$, $T = \frac{2\pi N}{\omega _A}$, *c* is the speed of light, $I_0$ the peak intensity, *N* the number of cycles in the pulse, $\varphi$ is the carrier-to-envelope phase (CEP) and $\omega _A$ denotes the central frequency of the vector potential which has been frequency corrected^41^ to produce an electric field with a central frequency $\omega$. The present calculations are performed utilizing single active electron potentials for He atom and Ne atom^42^ with the electron initially prepared in a superposition of the ground and an excited state. The TDSE has been solved by expanding the wavefunction in spherical harmonics (up to $\ell _{max} = 50$ for all relevant *m* values), as described in^27^. The computations have been performed on a radial grid of 300 a.u. with a grid spacing of 0.05 a.u.  using exterior complex scaling on the outer 15 a.u. of the grid. A time step of 0.05 a.u. has been used.

## Results and discussion

**Figure 3:**
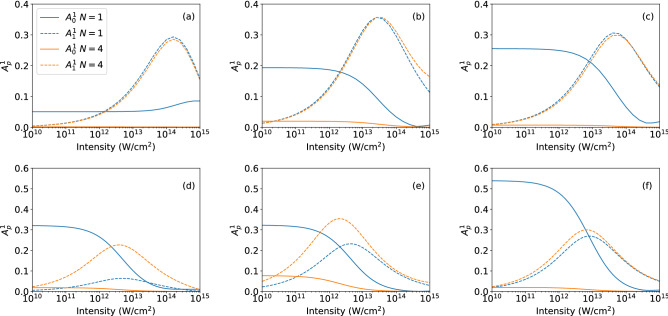
Generalized asymmetry parameters $A_0^1$ (solid lines) and $A_1^1$ (dashed lines) as function of intensity for ionization of superpositions of $2p_{-1}-3d_0$ (a), $2p_{0}-3d_1$ (**b**), and $2p_{1}-3d_2$ (**c**) in neon atom with initial populatations of 0.5 for each state (top row). Results in bottom row for superpositions with populations of $P_{2p_{-1}}=0.98,P_{3d_0}=0.02,P_{4s_0}=0.00057$ (**d**), $P_{2p_{0}}=0.94,P_{3d_1}=0.06$ (**e**), and $P_{2p_{1}}=0.88,P_{3d_2}=0.12$ (**f**). Results have been obtained for one-cycle (blue lines) and four-cycle (orange lines) pulses at photoelectron energy $E = 2\omega -I_p$.

**Figure 4:**
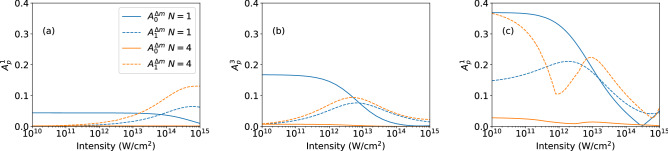
Same as Fig. 3 but for superpositions generated by a right-handed circularly polarized pulse via one-photon transition in helium atom (**a**), three-photon transition in neon atom (**b**), and one-photon transition in neon atom (**c**) (for details see text).

For our applications we have considered individual superposition states in neon (Fig. 3) and realistic superposition states considering all possible initial $m_g$ states of neon and helium atoms (Fig. 4). The central frequency of the applied electric field was set to the energy difference of the initially populated field-free states ($\omega _0=|E_g-E_e|$). To analyze the relevance of both the short-pulse parameter $A_0^{\Delta m}$ and the high-intensity parameter $A_1^{\Delta m}$ calculations have been performed for one- and four-cycle probe pulses (FWHM pulse durations) as function of the peak intensity of the pulse. At the end of each simulation of the time-dependent Schrödinger equation we obtained the photoelectron angular distribution at a given momentum *k* (corresponding to a photoelectron energy of $E=2\omega -|E_g|$) by projecting the wavefunction onto the field-free continuum states on the numerical grid. The asymmetry parameters $A_0^{\Delta m}$ and $A_1^{\Delta m}$ are then determined from the numerical PADs using Eq. (5).

To exemplify the application and provide basic insights in the relevance of the GAPs, we have first considered superpositions of individual sublevels in different shells of the neon atom as initial state. Figure 3 shows results for the GAPs as a function of peak intensity for interaction of a neon atom, prepared in $(2p_{-1}-3d_0)$ (a,d), $(2p_{0}-3d_1)$ (b, e), and $(2p_{1}-3d_2)$ (c, f) superpositions. For the results in the top row equal population in the two initial states has been considered, while for those in the bottom row populations, as produced via one-photon excitation by a 50 cycle, $10^{13}$ W/$\hbox {cm}^2$ right handed circulary polarized pump pulse, are used for the initial state (states with populations larger than $10^{-5}$ are considered, see figure caption for values). The central frequency of the ionizing probe laser pulse is tuned to the energy gap between the field-free ground and excited states.

Overall, except for the results in panel (a) which we will discuss below separately, the short-pulse parameter $A_0^1$ (solid lines) is large at low intensities and for the shorter of the two pulse durations. This is expected from our discussion above and confirms that the parameter is an indicator for the interference of the one-photon signal from the ground state and the one-photon signal from the excited state. Since both of these signals are first order in intensity, their relative strengths and, hence, the $A_0^1$ parameter are independent of intensity at low intensities for a given initial superposition state. As the intensity increases, the relative amplitude of the two-photon ionization channel increases and finally dominates over the one-photon channel from the ground state. This trend is reflected in both the short-pulse and the high-intensity parameters. The increase in $A_1^1$, resulting from the interference between the one-photon excited state signal and the two-photon ground state signal, shows in which intensity regime the two-photon signal starts to overtake the short pulse signal. Simultaneously with the increase of $A_1^1$ we observe a decrease of the $A_0^1$ signal, in agreement with our physical interpretation of the relevance of the different pathways. Parenthetically, we note the onset of an increase of the $A_0^1$ parameter at high peak intensities is likely due to pathways involving higher order processes with an even number of total photons.

While the general trends of the two parameters are similar in most of the cases, there is one exception where the details differ significantly. In the case of the $(2p_{-1}-3d_0)/\sqrt{2}$ superposition (panel a), $A_0^1$ shows a different trend. It remains constant at low intensities before increasing at higher intensities. As the laser pulse and relative populations are fixed in the top row (a–c) of Fig. 3, the change in amplitude and shape of $A_0^1$ and $A_1^1$ are caused by changes in the cross-sections of the various states. We, therefore, attribute the different trend in the $A_0^1$ signal in panel (a) to be due to the dominance of the one-photon transition from the $3d_0$ state at all intensities, since $m=0$ states are, in general, easier to ionize with a linearly polarized pulse than those with $|m| > 0$. This interpretation is further supported by the results in Fig. 3d for a superposition with much lower initial population in the $3d_0$-state. Despite the difference in magnitude of transition amplitudes, now the interference between the one-photon pathways is effective and the general expected trend for the short-pulse parameter $A_0^1$ is present.

Comparison of the results in the two rows of Fig. 3 shows that the intensity at which the transitions in the parameters occur depends on the population in the two states in the initial superposition. Since in the bottom row the populations in the excited states are low, the relative magnitudes of the corresponding one-photon transition amplitudes from the excited states as compared to those from the ground state levels are weaker than for the equally populated superpositions (top row). Therefore, we observe the impact of the two-photon pathway from the ground state at lower intensities in the results in the bottom row. Additionally, the $A_1^1$ signal becomes sensitive to changes in pulse length due to the weak signal from the excited state. Thus, the GAPs may also be useful to detect the population ratio in the superposition states. Here, we do not further analyze this feature, but focus on the more general application of the parameters to identify the presence of different ionization pathways.

So far, in Fig. 3 we considered simple albeit somehow artificial superposition states restricted to certain sublevels of two shells in the neon atom. Now, we extend our analysis to three different initial states as they can be generated with right-handed circularly polarized pump laser pulses: (a) one-photon excitation of helium atom to the $2p_1$ state leading to $(1s-2p_1)/\sqrt{2}$ superposition, (b) three-photon excitation of neon atom to the 3*d* shell, leading to an initial state consisting of a superposition with equal population in $2p_{-1}$ and $3d_2$ along with populated $2p_0$, and $2p_1$ states (note, that the latter two 2*p* states cannot be excited by the absorption of three right-handed circularly polarized photons to the 3*d* shell), and (c) one-photon excitation of neon atom to the 3*d* shell leading to the combination of the three superposition states analyzed separately in the bottom row of Fig. 3. In Fig. 4, we present results for the GAPs of the corresponding calculations. The initial superpositions in case of the helium data (a) and the neon data in panel (b) consist of just one state in the excited shell and show the same general trends as those in the majority of the panels of Fig. 3 with the decrease of $A_0^{\Delta m}$ along with the increase of $A_1^{\Delta m}$ occurring in a certain intensity regime. The transitions in helium occur at a higher peak intensity due to the more tightly bound helium ground state, which is harder to ionize. The overall trends of the results in Fig. 4c for the more complex initial superposition, consisting of six states in the 2*p*- and the 3*d*-shell of neon, are the same as before. The one-cycle pulse data are similar to those discussed before, providing the same information about the impact of the two-photon transition from the ground state. However, the $A_1^1$ parameter, particularly at the longer pulse duration, shows an additional interference structure in the transition regime. It is likely that this is due to an interference between transitions originating from two excited states differing by $\Delta m = 1$, here between one-photon ionization signals from $3d_0$ and $3d_1$ as well as from $3d_1$ and $3d_2$. Although the number of absorbed photons in these transition is not 3, the resulting $\gamma$ has the same parity as the signal from a $2p_m-3d_{m+1}$ state.

**Figure 5:**
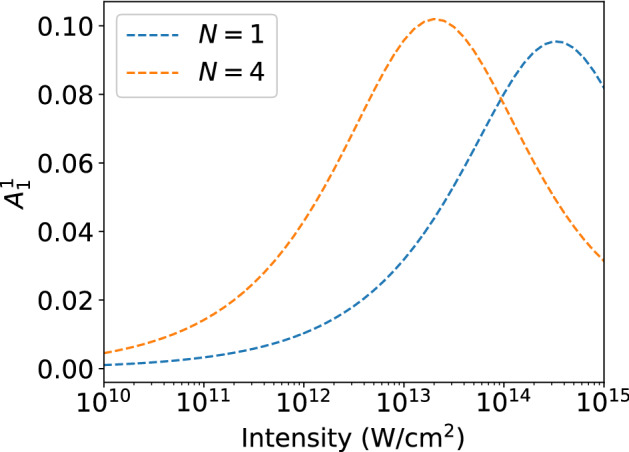
Asymmetry parameter $A_1^1$ for ionization of helium atom (1*s*–$2p_1$) with one- and four-cycle pulses at central frequency $\omega =1.2\omega _0$.

The high-intensity asymmetry parameter $A_1^{\Delta m}$ is not only indicative of the general features concerning the interferences between the pathways discussed above, but also provides insights in more subtle aspects. In the top row of Fig. 3 the maximum of the asymmetry parameter $A_1^{\Delta m}$ occurs at the same intensity independent of the pulse duration. This is due to the fact that in the calculations the central frequency was set to the energy difference of the initially populated states. In this case the center of the photoelectron energy distributions due to the one-photon ionization from the excited state and the two-photon transition from the ground state nearly coincide^43^. If the frequency is detuned from the resonance, the position of maximum interference strongly depends on the pulse duration. Due to the larger spectral width of the pulse at shorter pulse duration, the interference between the channels becomes most effective at higher intensities than the longer pulse duration results, at which the photoelectron distributions from the two channels have less overlap. This effect is seen in the results in Fig. 5 for photoionization at a central frequency of 1.2 times the energy difference between the 1*s*- and $2p_1$-states in helium atom.

**Figure 6:**
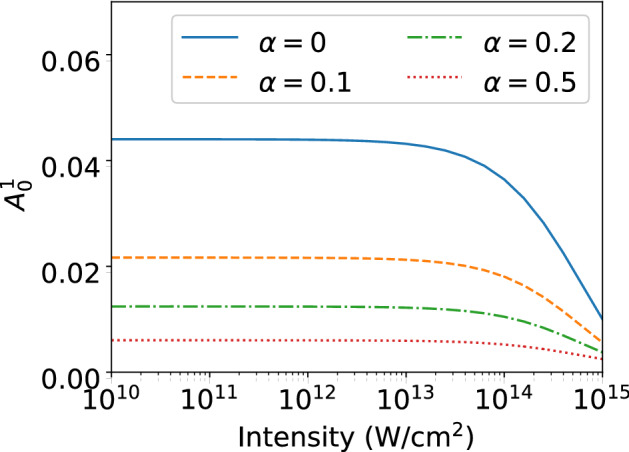
Asymmetry parameter $A_0^1$ for ionization of helium atom (1*s*–$2p_1$) with one-cycle pulses at central frequency $\omega _0$. Results of averages over different Gaussian distributions of CEP with width $\alpha$ are compared with those at fixed CEP ($\alpha =0$, solid line).

Finally, it is important to consider variations in the laser parameters relevant for an application of the generalized asymmetry parameters in an experiment. Typically, the peak intensity of the applied laser pulse may vary from shot to shot as well as over the interaction volume. The results in Figs. 3 and 4 show that the two GAPs vary rather slowly as a function of peak intensity. Thus, we can expect that variations in intensity in an experiment will not impact the results significantly. Another parameter that is usually difficult to control is the CEP of a laser pulse, which typically may become most important for ultrashort pulses. In the present study the high-intensity $A_1^{1}$ parameter is independent of variations in CEP. To study the dependence on the CEP for the short-pulse low-intensity asymmetry parameter $A_0^{1}$ we have obtained results as a function of the CEP and averaged the results about a given value $\varphi _0$ using a Gaussian distribution of width $\alpha$ (in units of $2\pi$) as:

8 $$\begin{aligned} A_{p}^{\Delta m}(I, T; \alpha ) = \frac{\sum _i \exp \left[ -\frac{1}{2}\left( \frac{|\varphi _i-\varphi _0|}{2\pi \alpha }\right) \right] \sigma (\varphi _i) A_{p}^{\Delta m}(\varphi _i,I,T) }{\sum _i\exp \left[ -\frac{1}{2}\left( \frac{|\varphi _i-\varphi _0|}{2\pi \alpha }\right) \right] \sigma (\varphi _i)}\; , \end{aligned}$$

where $\sigma (\varphi _i)$ is the total ionization probability at CEP $\varphi _i$. In the calculations we have assumed that the pump pulse (for the preparation of the initial state) has the same CEP as the probe pulse. The comparison of the results for different averages in Fig. 6 with the results for fixed CEP (solid line) shows that for He atom the short-pulse asymmetry parameter $A_0^1$ is indicative for the interference up to fluctuations of about $\pi /2$ in the CEP.

## Summary

In summary, we have introduced a set of generalized asymmetry parameters (GAPs) which characterize the interference of linear and nonlinear pathways to ionization of atoms, prepared in superposition states, due to the interaction with a linearly polarized intense ultrashort laser pulse. These parameters may provide a new tool to analyze data in attosecond experiments. The relevance of the parameters is demonstrated via the results of numerical simulations of ionization of helium and neon atom. The impact of short pulse and nonlinear effects, as they arise in experiments with free-electron lasers and high-order harmonic generation, is shown. The dependence on the central frequency of the applied laser pulse and the impact of variations of laser parameters, such as the peak intensity and the carrier-to-envelope phase, are analyzed and discussed.

## References

[1] Seddon EA (2017). Short-wavelength free-electron laser sources and science: a review. Rep. Prog. Phys..

[2] Popmintchev T, Chen M-C, Arpin P, Murnane MM, Kapteyn HC (2010). The attosecond nonlinear optics of bright coherent X-ray generation. Nat. Photonics.

[3] Chini M, Zhao K, Chang Z (2014). The generation, characterization and applications of broadband isolated attosecond pulses. Nat. Photonics.

[4] Zhao K (2012). Tailoring a 67 attosecond pulse through advantageous phase-mismatch. Opt. Lett..

[5] Chen M-C (2014). Generation of bright isolated attosecond soft X-ray pulses driven by multicycle midinfrared lasers. Proc. Natl. Acad. Sci..

[6] Vrakking MJJ (2014). Attosecond imaging. Phys. Chem. Chem. Phys..

[7] Pazourek R, Nagele S, Burgdörfer J (2015). Attosecond chronoscopy of photoemission. Rev. Mod. Phys..

[8] Calegari F, Sansone G, Stagira S, Vozzi C, Nisoli M (2016). Advances in attosecond science. J. Phys. B At. Mol. Opt. Phys..

[9] Xu J, Blaga CI, Agostini P, DiMauro LF (2016). Time-resolved molecular imaging. J. Phys. B At. Mol. Opt. Phys..

[10] Ramasesha K, Leone SR, Neumark DM (2016). Real-time probing of electron dynamics using attosecond time-resolved spectroscopy. Annu. Rev. Phys. Chem..

[11] Peng P, Marceau C, Villeneuve DM (2019). Attosecond imaging of molecules using high harmonic spectroscopy. Nat. Rev. Phys..

[12] Cavalieri AL (2007). Attosecond spectroscopy in condensed matter. Nature.

[13] Schultze M (2010). Delay in photoemission. Science.

[14] Klünder K (2011). Probing single-photon ionization on the attosecond time scale. Phys. Rev. Lett..

[15] Tao Z (2016). Direct time-domain observation of attosecond final-state lifetimes in photoemission from solids. Science.

[16] Isinger M (2017). Photoionization in the time and frequency domain. Science.

[17] Remetter T (2006). Attosecond electron wave packet interferometry. Nat. Phys..

[18] Mauritsson J (2010). Attosecond electron spectroscopy using a novel interferometric pump-probe technique. Phys. Rev. Lett..

[19] Gruson V (2016). Attosecond dynamics through a Fano resonance: monitoring the birth of a photoelectron. Science.

[20] Ishikawa KL, Ueda K (2012). Competition of resonant and nonresonant paths in resonance-enhanced two-photon single ionization of he by an ultrashort extreme-ultraviolet pulse. Phys. Rev. Lett..

[21] Ma R (2013). Photoelectron angular distributions for the two-photon ionization of helium by ultrashort extreme ultraviolet free-electron laser pulses. J. Phys. B At. Mol. Opt. Phys..

[22] Grum-Grzhimailo AN, Gryzlova EV, Staroselskaya EI, Venzke J, Bartschat K (2015). Interfering one-photon and two-photon ionization by femtosecond VUV pulses in the region of an intermediate resonance. Phys. Rev. A.

[23] Douguet N (2016). Photoelectron angular distributions in bichromatic atomic ionization induced by circularly polarized VUV femtosecond pulses. Phys. Rev. A.

[24] Hofbrucker J, Volotka A, Fritzsche S (2018). Maximum elliptical dichroism in atomic two-photon ionization. Phys. Rev. Lett..

[25] Boll DIR, Fojón OA, McCurdy CW, Palacios A (2019). Angularly resolved two-photon above-threshold ionization of helium. Phys. Rev. A.

[26] Wang M-X, Liang H, Xiao X-R, Chen S-G, Peng L-Y (2019). Time-dependent perturbation theory beyond the dipole approximation for two-photon ionization of atoms. Phys. Rev. A.

[27] Venzke J, Jaroń-Becker A, Becker A (2020). Ionization of helium by an ultrashort extreme-ultraviolet laser pulse. J. Phys. B At. Mol. Opt. Phys..

[28] Yin Y-Y, Chen C, Elliott DS, Smith AV (1992). Asymmetric photoelectron angular distributions from interfering photoionization processes. Phys. Rev. Lett..

[29] Böwering N (2001). Asymmetry in photoelectron emission from chiral molecules induced by circularly polarized light. Phys. Rev. Lett..

[30] Ilchen M (2018). Symmetry breakdown of electron emission in extreme ultraviolet photoionization of argon. Nat. Commun..

[31] Cirelli C (2018). Anisotropic photoemission time delays close to a Fano resonance. Nat. Commun..

[32] Maulbetsch F, Briggs JS (1992). Asymmetry parameter for double photoionization. Phys. Rev. Lett..

[33] Garcia GA, Nahon L, Daly S, Powis I (2013). Vibrationally induced inversion of photoelectron forward-backward asymmetry in chiral molecule photoionization by circularly polarized light. Nat. Commun..

[34] Prince KC (2016). Coherent control with a short-wavelength free-electron laser. Nat. Photonics.

[35] Chelkowski S, Bandrauk AD (2002). Sensitivity of spatial photoelectron distributions to the absolute phase of an ultrashort intense laser pulse. Phys. Rev. A.

[36] Saffman M (2016). Quantum computing with atomic qubits and Rydberg interactions: progress and challenges. J. Phys. B At. Mol. Opt. Phys..

[37] Goulielmakis E (2010). Real-time observation of valence electron motion. Nature.

[38] Holler M, Schapper F, Gallmann L, Keller U (2011). Attosecond electron wave-packet interference observed by transient absorption. Phys. Rev. Lett..

[39] Xie X (2012). Attosecond probe of valence-electron wave packets by subcycle sculpted laser fields. Phys. Rev. Lett..

[40] In this work we consider the case that ground and excited state have different magnetic quantum numbers ($$m_g \ne m_e$$). We note that for $$m_g=m_e$$ only the asymmetry parameters for odd $$\gamma $$ are applicable.

[41] Venzke J, Joyce T, Xue Z, Becker A, Jaron-Becker A (2018). Central frequency of few-cycle laser pulses in strong-field processes. Phys. Rev. A.

[42] Reiff R, Joyce T, Jaron-Becker A, Becker A (2020). Single-active electron calculations of high-order harmonic generation from valence shells in atoms for quantitative comparison with TDDFT calculations. J. Phys. Commun..

[43] The small discrepancy is due to the energy dependence of the one- and two-photon cross sections.

